# Dengue emergence risk in Tehran, Iran: An integrative review of *Aedes* ecology, climate, urban environmental determinants, and public health preparedness

**DOI:** 10.1016/j.puhip.2026.100878

**Published:** 2026-10-31

**Authors:** Ebrahim Abbasi, Asghar Talbalaghi

**Affiliations:** a Italian Mosquito Control Association, Comune di Alessandria, ALESSANDRIA, PIEDMONT, Italy; b Iran University of Medical Sciences, Tehran, Iran

**Keywords:** Dengue, *Aedes aegypti*, *Aedes albopictus*, Tehran, Environmental receptivity, Climate change, Vector ecology, Vector surveillance, Public health preparedness

## Abstract

**Objectives:**

To synthesize the available evidence concerning *Aedes* mosquito ecology and potential establishment, climatic and environmental suitability, epidemiological and urban determinants, and public health surveillance and preparedness relevant to dengue emergence in Tehran, Iran. The review also aimed to distinguish environmental receptivity and potential vector establishment from documented autochthonous dengue transmission and to identify key evidence gaps and surveillance priorities.

**Study design:**

Structured narrative/integrative review.

**Methods:**

A structured literature search was conducted covering publications and relevant publicly available information from January 2000 to March 2025. PubMed, Scopus, Web of Science, ScienceDirect, Google Scholar, and selected Iranian institutional sources were searched.

**Evidence was synthesized across four predefined domains:**

*Aedes* vector ecology and potential establishment; climatic and environmental suitability; epidemiological, population-level, urban, and infrastructural determinants; and vector surveillance, control, and public health preparedness. Twelve core studies were selected for focused qualitative and integrative analytical synthesis. Evidence was interpreted according to its geographical relevance and methodological basis, distinguishing direct Tehran-specific findings from Iranian, regional, international, and model-based evidence.

**Results:**

Available evidence did not demonstrate sustained establishment of *Aedes* aegypti or Aedes albopictus populations or sustained autochthonous dengue transmission in Tehran. However, climatic variability, warming trends, urban environmental conditions, potential breeding habitats, population mobility, and regional entomological evidence indicate potentially relevant conditions for future environmental receptivity. Climate and spatial models suggest possible expansion of environmental suitability for *Aedes* vectors and dengue-related transmission under warming scenarios, although these projections do not establish local vector populations or demonstrate dengue transmission in Tehran. Important limitations in standardized longitudinal entomological surveillance and integrated epidemiological and laboratory monitoring remain.

**Conclusions:**

Tehran is best characterized as a setting of potential and evolving dengue receptivity rather than established dengue transmission. The available evidence does not justify concluding that persistent *Aedes* populations or sustained local dengue transmission are established in the city. Standardized longitudinal entomological surveillance, integrated epidemiological and laboratory monitoring, environmental assessment, and coordinated public health preparedness are warranted to reduce uncertainty and facilitate early detection of potential dengue emergence.

## Graphical abstract

**Figure:**
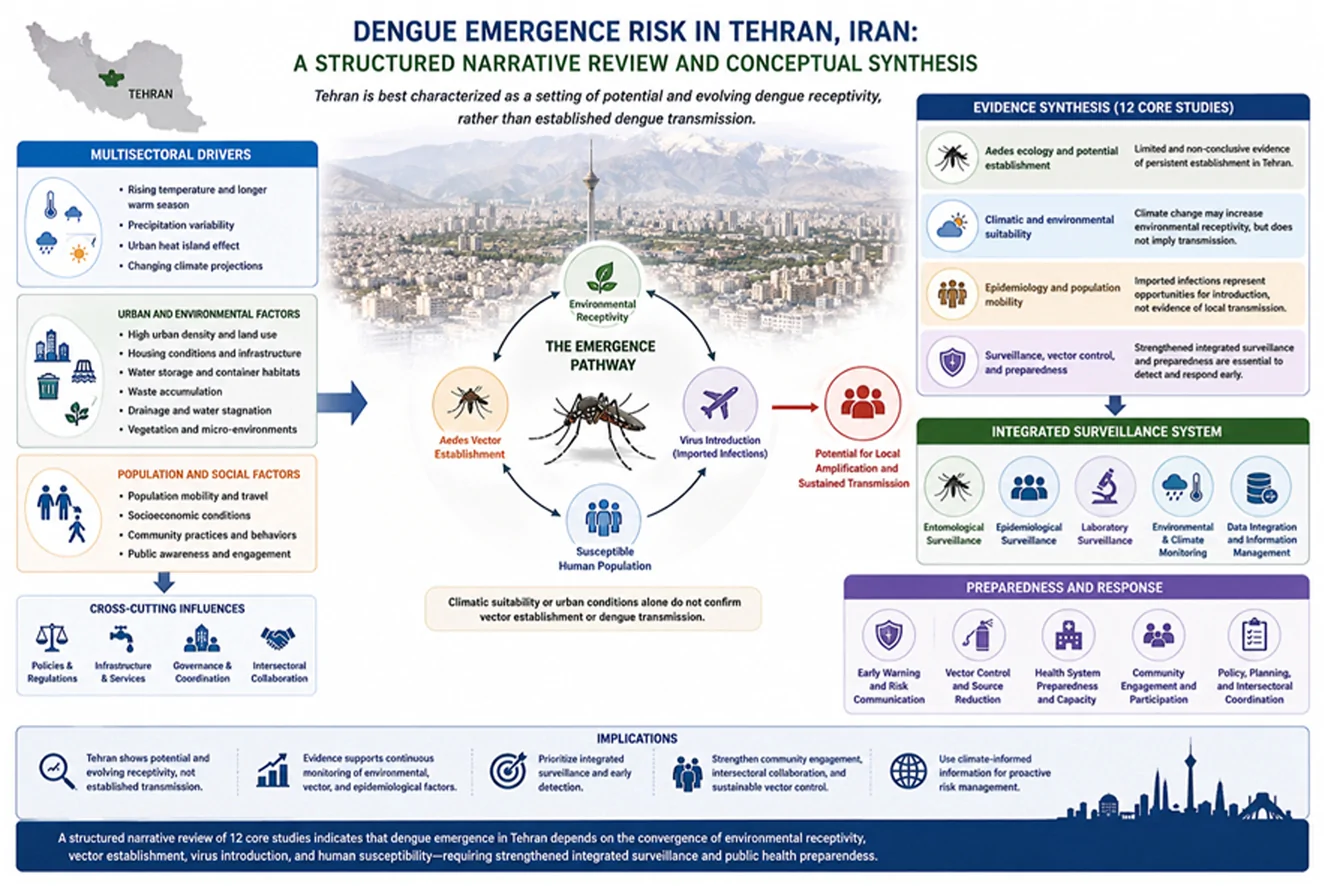

## 1 Introduction

Dengue is a major mosquito-borne viral disease of global public health importance, with its epidemiological distribution closely linked to the ecology, geographic range, seasonal activity, and population dynamics of competent *Aedes* mosquito vectors. The principal vectors, *Aedes aegypti* and *Aedes albopictus*, are particularly well adapted to human-dominated environments and can exploit artificial containers, household water-storage facilities, discarded materials, and other small aquatic habitats that may occur within densely populated urban areas. The ecological suitability of these vectors is influenced by multiple interacting factors, including temperature, precipitation, humidity, availability and persistence of breeding habitats, vegetation and land-use patterns, housing characteristics, human behavior, and population mobility. Consequently, the emergence of dengue in an urban setting where sustained local transmission has not previously been established should not be inferred from any single environmental or epidemiological indicator. Rather, dengue emergence represents a multistage process requiring an appropriate ecological environment, the presence or establishment of competent vectors, introduction of dengue virus, sufficient opportunities for human–vector contact, and adequate temporal and spatial overlap among these components. This distinction is particularly important when assessing cities in which evidence of vector establishment or autochthonous transmission remains limited, because climatic or environmental suitability may indicate receptivity to emergence without demonstrating that transmission is occurring or will necessarily occur [1], [2], [3], [4].

Iran provides an important context in which these relationships can be examined because the country is not currently characterized by established endemic dengue transmission, while evidence relevant to *Aedes* ecology and arboviral surveillance has emerged from several regions. Entomological and arbovirus-related investigations conducted in Iran, particularly outside Tehran, have documented mosquito fauna, ecological conditions, and surveillance considerations that are relevant to understanding the potential distribution and adaptation of competent vectors. In addition, evidence from regional and international settings indicates that *Aedes* mosquitoes can persist across a broader range of environmental conditions than traditionally associated with tropical dengue transmission. However, evidence generated outside Tehran cannot be interpreted as direct evidence of persistent *Aedes aegypti* or *Aedes albopictus* establishment within the capital. The available evidence reviewed for Tehran does not demonstrate sustained establishment of either species or sustained autochthonous dengue transmission. At the same time, the absence of sufficiently comprehensive longitudinal entomological surveillance should not be interpreted as definitive evidence that transient introductions or geographically restricted vector populations could never occur. The appropriate interpretation is therefore one of uncertainty: available evidence does not establish ongoing local transmission, but important ecological, environmental, and epidemiological conditions warrant assessment because they could modify the city's future receptivity to dengue emergence [5], [6], [7], [8].

Tehran is particularly relevant to this assessment because of its large and densely populated metropolitan structure and its substantial spatial heterogeneity in urban form, land use, housing conditions, water-management practices, environmental maintenance, and population density. Urbanization can modify local climatic and ecological conditions and can generate heterogeneous microenvironments that influence mosquito survival, reproduction, and access to aquatic breeding habitats. Potentially relevant features include household and community water-storage practices, artificial containers, localized waste accumulation, drainage conditions, housing characteristics, and other human-generated environments capable of retaining water. These characteristics should not, however, be interpreted individually as evidence of *Aedes* establishment. Rather, they represent components of environmental receptivity that could become epidemiologically relevant if competent vectors are introduced and subsequently persist. The heterogeneity of these conditions also means that city-wide meteorological measurements may not adequately capture the microenvironmental variation relevant to mosquito ecology. Accordingly, assessment of dengue emergence risk in Tehran requires consideration of environmental and urban determinants at an appropriate spatial scale while avoiding the assumption that the presence of potentially suitable breeding environments necessarily indicates an established vector population. The currently available evidence does not support a validated quantitative estimate of the probability of *Aedes* establishment at the district level, and the relative contribution of individual urban determinants remains insufficiently quantified for Tehran [3], [6], [9], [10].

Climate represents another important component of the ecological pathway linking environmental conditions to potential dengue emergence. Temperature can influence mosquito development, survival, feeding behavior, reproductive activity, and the biological processes associated with pathogen development within the vector, while precipitation and water availability can affect the formation and persistence of aquatic breeding habitats. Evidence from ecological and climate-based studies has suggested that warming and other climatic changes may alter the geographic and seasonal suitability of environments for *Aedes* mosquitoes. Global and regional modeling studies have likewise projected changes in areas that may be environmentally suitable for *Aedes* vectors and dengue transmission under future warming scenarios. Such evidence is relevant to Tehran because it provides a plausible mechanism through which environmental receptivity could change over time. Nevertheless, climatic suitability is not equivalent to vector establishment, and projected climatic suitability should not be interpreted as a quantitative prediction of dengue transmission in Tehran. The reviewed evidence does not provide a Tehran-specific dynamically downscaled climate–dengue transmission model capable of estimating future dengue incidence attributable to climate change. Therefore, climatic observations and model-based projections are considered in this review as contextual evidence concerning potential environmental suitability rather than direct evidence of local vector establishment or transmission. This distinction is essential for preventing an inappropriate transition from evidence of changing climatic conditions to an assumption of future dengue endemicity [11], [12], [13], [14].

The potential for dengue emergence also depends on epidemiological and population-level processes that extend beyond vector ecology and climate. Population mobility may provide opportunities for dengue virus introduction into receptive urban environments through travel-associated or otherwise imported infections. However, the occurrence of imported dengue infection represents an opportunity for viral introduction and does not itself demonstrate subsequent local transmission. For local transmission to become established, virus introduction must coincide with the presence of competent and sufficiently abundant vectors, appropriate human–vector contact, and environmental conditions that permit continued transmission. Social and infrastructural characteristics may modify these interactions through their effects on water management, sanitation, waste accumulation, housing conditions, environmental maintenance, community practices, and patterns of human–vector contact. These determinants may be spatially heterogeneous and may interact with climatic and ecological conditions, potentially producing localized differences in environmental receptivity. Nevertheless, the evidence currently available for Tehran is insufficient to quantify the independent contribution of these factors or to derive a validated district-level dengue emergence risk estimate. Their relevance should therefore be interpreted within an integrated ecological and public health framework rather than as independently demonstrated predictors of transmission [4], [15], [16], [17], [18].

Finally, the assessment of potential dengue emergence in Tehran requires consideration of surveillance and public health preparedness because the ability to detect an introduced vector or imported infection may substantially influence the opportunity for early intervention. Conventional mosquito-control activities do not necessarily provide sufficient longitudinal sensitivity to distinguish transient introductions from persistent *Aedes* populations, particularly when surveillance is not standardized with respect to sampling methods, geographic coverage, temporal repetition, larval surveillance, and species confirmation. The available evidence therefore supports the need for integrated entomological, epidemiological, environmental, and laboratory surveillance capable of identifying changes in vector distribution, detecting imported dengue infections, and clarifying whether environmental receptivity is translating into vector establishment or local transmission. Importantly, the present evidence base does not justify assigning a validated quantitative preparedness score to Tehran or specifying a particular trap density, surveillance frequency, or operational threshold as an empirically established requirement. Such recommendations should be distinguished from published guidance and, where not directly supported by external standards, should be presented as evidence-informed operational considerations derived from the synthesis. Against this background, the present study was undertaken as a structured narrative/integrative review to synthesize heterogeneous evidence concerning *Aedes* ecology and potential establishment, climatic and environmental receptivity, epidemiological and population-level determinants, and surveillance and public health preparedness relevant to dengue emergence in Tehran. Particular emphasis was placed on distinguishing evidence directly pertaining to Tehran and Iran from regional, international, and model-based evidence, and on maintaining a clear conceptual separation between environmental receptivity, potential vector establishment, and demonstrated dengue transmission. The objective was therefore not to estimate a quantitative probability of future dengue transmission, but to integrate the available evidence and identify the conditions, uncertainties, and surveillance priorities relevant to assessing whether Tehran's currently plausible pathway toward dengue emergence represents an established phenomenon or remains a potential future risk [18], [19], [20], [21].

## 2 Methods

### 2.1 Review design and rationale

This study was designed as a structured narrative/integrative review to synthesize heterogeneous evidence relevant to dengue emergence risk in Tehran, Iran. The review was intentionally framed as an integrative narrative synthesis rather than a conventional systematic review or meta-analysis because the available evidence encompasses substantially different study designs, geographical settings, population and environmental contexts, outcome measures, and methodological approaches. The evidence base includes entomological investigations, arbovirus surveillance studies, ecological and climatic assessments, regional and global climate-risk or spatial modeling studies, epidemiological evidence, and literature addressing vector surveillance, control, and public health preparedness. These sources address different levels of inference and cannot appropriately be treated as a homogeneous evidence base from which a pooled effect estimate or quantitative measure of dengue transmission risk can be derived. The purpose of the review was therefore to integrate complementary evidence streams and to characterize the conditions, uncertainties, and evidence gaps relevant to potential dengue emergence in Tehran rather than to calculate a pooled estimate of disease risk. The analytical framework was defined a priori around four interconnected domains [1]: *Aedes* vector ecology and the potential for vector establishment [2]; climatic and environmental suitability and receptivity [3]; epidemiological, population-level, urban, and infrastructural determinants relevant to dengue emergence; and [4] vector surveillance, vector-control capacity, and public health preparedness. Throughout the review, environmental receptivity, vector establishment, and dengue transmission were treated as distinct levels of inference. Environmental receptivity was defined as the presence of climatic, ecological, urban, or social conditions that could potentially support a competent vector if introduced. Vector establishment was considered to require evidence consistent with persistence and reproduction of a vector population over time and across an appropriate spatial context. Sustained dengue transmission was considered a further epidemiological state requiring virus introduction, competent and sufficiently abundant vectors, suitable human–vector interactions, and appropriate temporal and spatial overlap. This framework was used to prevent climatic or ecological suitability, isolated vector observations, or imported infections from being interpreted as evidence of established local transmission. The reporting structure was designed to improve transparency and traceability of the review process, with PRISMA-related reporting elements used where applicable to describe literature identification and selection. PRISMA 2020 was not used to redefine the study as a systematic review, because the present work was designed and conducted as an integrative narrative synthesis rather than a formal systematic review with quantitative evidence aggregation. This distinction is important because PRISMA is primarily a reporting framework and does not itself determine the underlying review design. The review was not prospectively registered, and no formal review protocol was registered before the literature search [22], [23], [24], [25].

### 2.2 Information sources and search strategy

The literature search covered publications and relevant publicly available information from January 2000 through March 2025. Multiple international, regional, and institutional sources were considered to reflect the multidisciplinary nature of dengue emergence and to reduce dependence on evidence from a single scientific discipline or geographical setting. The principal bibliographic sources were PubMed, Scopus, Web of Science, ScienceDirect, and Google Scholar, supplemented by selected Iranian institutional and publicly available sources considered relevant to mosquito ecology, arboviral surveillance, environmental conditions, and public health preparedness. The use of multiple sources was particularly important because evidence directly addressing dengue emergence in Tehran is limited, whereas potentially informative evidence is distributed across entomological, epidemiological, climatic, environmental, and public health literature. The search strategy combined terms representing dengue, competent mosquito vectors, geographical setting, environmental determinants, and public health context. Core search terms included “Dengue Fever,” “*Aedes aegypti*,” “*Aedes albopictus*,” “Tehran,” “Iran,” “climate change,” “vector-borne diseases,” “urban health,” “arboviruses,” and “public health preparedness.” These terms were combined using Boolean operators and adapted as necessary to the search characteristics of individual databases and information sources. The search was intentionally designed to identify not only studies directly examining dengue but also evidence concerning the ecological, climatic, environmental, epidemiological, and operational conditions that could influence potential dengue emergence in Tehran. Consequently, literature addressing *Aedes* ecology, climatic suitability, environmental conditions, vector surveillance, arboviral preparedness, and vector-control capacity was considered when its findings could reasonably contribute to interpretation of the Tehran context [8], [13], [26], [27], [28].

### 2.3 Eligibility criteria and geographic relevance

Records were considered potentially eligible when they addressed at least one of four predefined evidence domains [1]: the presence, ecology, distribution, or potential establishment of *Aedes aegypti* or *Aedes albopictus* in Tehran, elsewhere in Iran, or geographically relevant surrounding regions [2]; dengue epidemiology or arboviral evidence relevant to Iran [3]; climatic, meteorological, ecological, or environmental determinants potentially affecting *Aedes* ecology, vector suitability, or dengue-related processes; or [4] prevention, vector-control, surveillance, or public health preparedness strategies applicable to metropolitan environments. This eligibility framework was intentionally broader than a dengue-only criterion because the principal analytical question concerned the conditions that could influence emergence in a city where sustained local dengue transmission has not been demonstrated. Thus, evidence concerning competent vectors and their ecology was considered relevant even when the individual study did not directly investigate dengue transmission. Geographical relevance was explicitly incorporated into interpretation. Evidence generated directly from Tehran was considered the most directly informative for the local assessment. Evidence from other regions of Iran or geographically relevant neighboring settings was treated as contextual evidence, while studies from comparable urban or climatic environments and broader regional or global datasets were used to inform interpretation of ecological mechanisms, climatic suitability, or preparedness considerations. Evidence originating outside Tehran was therefore not treated as direct evidence of conditions within the city. In particular, regional or global model projections were interpreted as evidence concerning potential environmental or climatic suitability rather than as evidence of *Aedes* establishment or dengue transmission in Tehran. This hierarchy of geographical relevance was maintained throughout data extraction and synthesis to minimize inappropriate extrapolation from distant settings. Editorials, letters, and publications lacking sufficient methodological information for meaningful interpretation were excluded from the analytical evidence base. Because the objective was integrative interpretation rather than estimation of a single quantitative outcome, eligibility was determined according to both geographical relevance and the ability of the evidence to contribute meaningfully to at least one of the predefined analytical domains. This approach was necessary because a substantial proportion of the literature relevant to Tehran derives from studies conducted elsewhere in Iran, neighboring or climatically comparable settings, and regional or global modeling studies [3], [4], [29], [30].

### 2.4 Study identification and selection

The study-selection process proceeded sequentially from identification and preliminary screening of potentially relevant records to title and abstract assessment and subsequent full-text assessment of records considered potentially eligible. Two reviewers independently assessed the available records against the predefined eligibility domains. Disagreements concerning interpretation or eligibility were resolved through discussion and consensus. This independent assessment and consensus procedure was intended to improve consistency in study selection while recognizing that the review involved heterogeneous evidence types that could not be reduced to a single quantitative eligibility criterion. The broader literature identification and eligibility process was distinguished from the focused analytical synthesis. Following assessment of the potentially relevant evidence, 12 core studies were selected for detailed analytical synthesis (Fig. 1). The selection of these 12 studies was based on their combined relevance to the principal questions of the review and their capacity to provide interpretable evidence concerning *Aedes* ecology and potential establishment, climatic and environmental suitability, climate-related changes, dengue or arboviral risk, and public health surveillance and preparedness. The 12 studies were deliberately selected as complementary evidence sources rather than as a statistically representative sample of the entire dengue literature. Their characteristics, geographical settings, study designs, thematic contributions, and principal findings relevant to Tehran are presented in Table 1. The focused synthesis therefore should not be interpreted as indicating that only 12 publications were identified during the broader search or that these 12 studies constitute the entirety of the available literature. Rather, they represent the subset considered most informative for structured analytical integration across the predefined domains. Their methodological heterogeneity was retained as an explicit feature of the evidence base. Iranian entomological and arbovirus-surveillance studies contributed relatively direct contextual information; international ecological studies provided evidence concerning vector adaptation and environmental determinants; climate-risk and spatial modeling studies informed interpretation of potential changes in environmental suitability; and broader public health literature contributed evidence concerning surveillance, vector control, and preparedness. This approach provided analytical depth while avoiding inappropriate quantitative pooling of fundamentally different evidence types [24], [25], [31], [32].

**Fig. 1:**
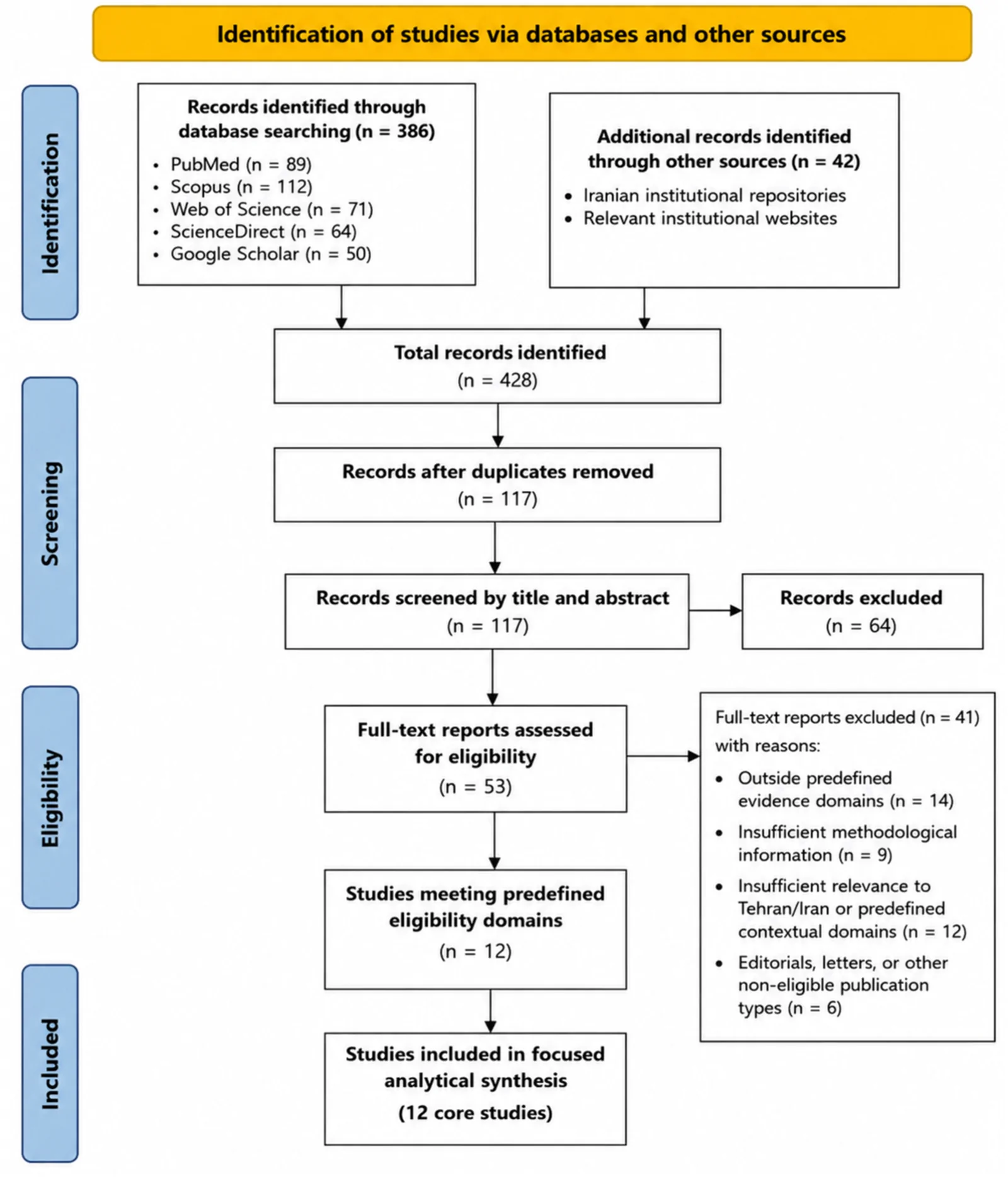
PRISMA-informed flow diagram of literature identification, screening, and selection. This PRISMA-informed flow diagram summarizes the identification, deduplication, screening, eligibility assessment, and final selection of evidence for the structured narrative/integrative review. A total of 420 records were identified, including 365 records retrieved through database searching and 55 records identified through additional sources. After removal of 122 duplicates, 298 records underwent title and abstract screening, of which 251 were excluded. Forty-seven full-text articles were subsequently assessed for eligibility, with 35 excluded for insufficient relevance, limited methodological information, inadequate comparability with the Tehran context or predefined evidence domains, or other eligibility-related reasons. Twelve studies were ultimately retained for quality assessment and included in the focused analytical synthesis. The flow diagram is PRISMA-informed and is intended to transparently report the study-selection process without implying that the review was conducted as a conventional systematic review or meta-analysis.

**Table 1:** Characteristics of the 12 core studies included in the focused integrative narrative synthesis.

Study/Reference	Geographic Context	Study Design/Type	Primary Focus	Key Findings	Relevance to Tehran	Role in the Present Synthesis
**Abedi-Astaneh et al. (2025)**[33]	Qom, central Iran	Field-based entomological surveillance with morphological and molecular identification and molecular arbovirus screening	Mosquito fauna and molecular detection of arboviruses	A total of 83,414 mosquitoes were collected during 2019–2020. *Culex tarsalis*, *Cx. theileri*, and *Cx. quinquefasciatus* were detected, including the first reported detection of *Cx. tarsalis* in Iran. No alphavirus, flavivirus, or phlebovirus infection was detected in the collected mosquitoes. The authors emphasized the importance of continued entomological and virological surveillance in the context of climatic and environmental change.	Indirect but Iran-specific. Provides contemporary evidence that mosquito composition can change within Iran and demonstrates the value of systematic entomological and molecular surveillance. It does not provide evidence of *Aedes aegypti* or *Ae. albopictus* establishment or dengue transmission in Tehran.	Supports the discussion of national entomological surveillance capacity, changing mosquito distributions, and the need for continued vector and arbovirus monitoring.
**Achee et al. (2015)**[21]	International/global	Critical public-health review and expert assessment of dengue vector-control interventions	Effectiveness, limitations, and integration of dengue vector-control approaches	No single vector-control intervention was considered sufficient for sustained dengue prevention. The review emphasized integrated, context-specific approaches, proactive control, operational and epidemiological surveillance, source reduction, community participation, and rapid response during outbreaks.	Indirect/contextual. Provides general evidence relevant to urban dengue preparedness and vector-control planning but does not evaluate interventions in Tehran.	Provides the vector-control and integrated-vector-management evidence base underlying the preparedness and response component of the review.
**Barrera et al. (2011)**[34]	San Juan, Puerto Rico	Longitudinal observational ecological study	Relationships among weather, human activities, *Aedes aegypti* abundance, oviposition, and dengue incidence	Weather variables and human activities influenced *Ae. aegypti* population dynamics. Human-managed containers were important larval habitats, and peaks in mosquito density preceded periods of maximum dengue incidence. Oviposition activity was associated with dengue incidence.	Indirect empirical evidence. Demonstrates how climatic variability and human-managed urban environments can jointly influence vector abundance and dengue dynamics; the relationships should not be interpreted as Tehran-specific transmission estimates.	Supports assessment of climatic variability, urban water-holding habitats, human behavior, vector abundance, and their potential interaction with dengue risk.
**Ebi and Nealon (2016)(13)**	Global	Climate–health review	Interactions among climate change, *Aedes* vectors, and dengue	The review identified climate as an important determinant of the geographic distribution and incidence of dengue and discussed how warming, together with globalization, travel, demographic change, and expanding vector ranges, may modify dengue risk.	Indirect/global evidence. Provides a mechanistic and conceptual basis for considering climate as a determinant of environmental receptivity, but does not establish a Tehran-specific climate–transmission relationship.	Provides the climate–dengue conceptual basis for interpreting climatic change as one component of potential future receptivity.
**Flacio et al. (2016)**[35]	Ticino, southern Switzerland	Longitudinal ecological surveillance with ovitrap monitoring and generalized linear mixed modeling	Introduction, spread, establishment, and climatic determinants of *Aedes albopictus*	Long-term surveillance documented progressive spread and local establishment of *Ae. albopictus*. Repeated positivity and increasing egg densities supported locally reproducing and overwintering populations. Temperature, precipitation, geographic position, and transport-related factors influenced spatial and temporal patterns.	Indirect but highly relevant ecological evidence. Demonstrates that repeated detection over time, reproductive evidence, and persistence are required to distinguish establishment from transient occurrence. It does not demonstrate establishment in Tehran.	Supports the critical distinction between sporadic detection, persistence, and confirmed vector establishment, particularly in temperate or transitional urban environments.
**Jaberhashemi et al. (2022)**[6]	Hormozgan Province, southern Iran	Field-based entomological survey	Culicinae species composition, diversity, abundance, and bionomics	The study collected 3236 larvae and 1901 adult mosquitoes representing 16 Culicinae species across four counties. It documented substantial ecological diversity and emphasized the importance of information on seasonality, host preference, and larval habitats for vector-management planning. The study was not a demonstration of persistent *Ae. aegypti* or *Ae. albopictus* establishment.	Regional Iran-specific evidence. Provides ecological and entomological context from southern Iran but should not be interpreted as direct evidence of the target Aedes species being established in Tehran.	Provides regional ecological context for mosquito fauna, habitat characteristics, seasonality, and the need for locally generated entomological evidence.
**Kraemer et al. (2019)**[3]	Global	Global spatial and predictive modeling	Historical and future geographic distribution of *Aedes aegypti* and *Ae. albopictus*	The study demonstrated that vector distributions are influenced by suitable climate and human movement and projected future changes in vector distributions under changing climate, urbanization, and connectivity. The study emphasized that projected suitable areas should not simply be assumed to become fully infested.	Indirect model-based evidence. Relevant for understanding potential changes in climatic and urban suitability and the role of connectivity and human movement, but not evidence of current or future establishment in Tehran.	Supports interpretation of climate, urbanization, connectivity, and human movement as determinants of potential vector distribution.
**Messina et al. (2019)**[4]	Global	Global predictive spatial modeling	Current and future environmental suitability and population at risk of dengue	Statistical mapping was used to estimate global environmental suitability for dengue and project future changes using climate, population, and socioeconomic scenarios. The study indicated potential expansion of areas and populations exposed to environmentally suitable conditions under changing environmental conditions.	Indirect global modeling evidence. Useful for contextualizing future dengue receptivity but cannot substitute for Tehran-specific entomological, epidemiological, or transmission data.	Supports the discussion of future environmental suitability and population exposure while reinforcing the distinction between modeled suitability and actual transmission.
**Parhizgari et al. (2021)**[8]	Iran	National narrative review	Epidemiology and challenges of vector-borne diseases in Iran	The review described the epidemiological context of vector-borne diseases in Iran and highlighted challenges associated with climate change, globalization, travel, vector adaptation, and the need for further surveillance and research.	Iran-specific contextual evidence. Relevant to the national setting and emerging vector-borne disease preparedness, although it does not provide longitudinal evidence of persistent *Aedes* establishment or dengue transmission in Tehran.	Provides national epidemiological and public-health context for interpreting emerging arboviral threats and surveillance needs in Iran.
**Rocklöv and Dubrow (2020)**[36]	Global	Expert commentary/perspective	Climate change and vector-borne disease prevention and control	The article emphasized that climate change is already affecting vector-borne disease transmission and geographic spread and argued for strengthened prevention and control measures as climatic conditions continue to change.	Indirect global evidence. Supports the broader rationale for climate-informed preparedness but does not provide Tehran-specific estimates of vector establishment or dengue transmission.	Supports the climate-change and public-health preparedness framework of the review.
**Ryan et al. (2019)**[14]	Global	Mechanistic temperature-dependent transmission modeling	Climate-driven expansion and redistribution of *Aedes*-borne virus transmission risk	The model estimated current and future temperature suitability for virus transmission by *Ae. aegypti* and *Ae. albopictus* under multiple climate scenarios. It projected geographic redistribution and, in many settings, expansion of seasonal transmission suitability under warming.	Indirect global model-based evidence. Relevant to potential climatic receptivity but does not demonstrate that Tehran currently supports persistent vectors or sustained dengue transmission. Model outputs should therefore not be interpreted as Tehran-specific transmission predictions.	Provides quantitative mechanistic context for the climate–temperature–Aedes transmission pathway and supports cautious interpretation of future climatic receptivity.
**Wilder-Smith et al. (2017)**[18]	International/global	Public-health perspective/review	Emerging arboviral diseases, surveillance, preparedness, research, and response	The authors emphasized the increasing importance of dengue, chikungunya, Zika, and other arboviral diseases in the context of urbanization, globalization, and international mobility. Strengthening surveillance, preparedness, research capacity, and coordinated public-health responses was identified as a priority.	Indirect but directly relevant to preparedness. Provides an international framework for strengthening surveillance and response to emerging arboviral threats, without providing Tehran-specific evidence.	Supports the integrated surveillance, early detection, preparedness, rapid response, and intersectoral coordination components of the proposed framework.

**Table note:** The 12 studies were retained for focused analytical synthesis because, collectively, they provide complementary evidence across four domains relevant to dengue emergence risk in Tehran [1]: mosquito ecology and potential vector establishment [2], climatic and environmental suitability [3], ecological, epidemiological, and population-related determinants, and [4] surveillance, vector control, preparedness, and response. The studies differ substantially in geographic setting, study design, outcome measures, and directness of evidence. Accordingly, their findings were synthesized qualitatively rather than quantitatively pooled. Evidence from southern and central Iran was treated as geographically contextual rather than as direct evidence for Tehran, while international and global studies were used to provide mechanistic, ecological, and public-health context. In particular, modeled climatic or environmental suitability was not interpreted as evidence of persistent *Aedes* establishment, and vector establishment was not assumed to imply sustained autochthonous dengue transmission in Tehran.

### 2.5 Data extraction and evidence characterization

Data were extracted using a standardized framework developed for the purposes of the review. Extracted information included study or report characteristics, geographical setting, study design, vector-related findings, relevant climatic and environmental variables, epidemiological implications, surveillance considerations, and public health implications. Particular attention was given to the geographical and evidentiary proximity of each finding to Tehran. Each relevant finding was considered in relation to whether it represented direct evidence from Tehran, evidence from another region of Iran, evidence from a comparable urban or climatic setting, or evidence derived from regional or global modeling. This distinction was maintained throughout the synthesis to avoid treating model-based or geographically indirect evidence as equivalent to direct entomological or epidemiological observations from Tehran. The evidence was organized according to four prespecified thematic domains [1]: vector ecology and potential establishment [2]; climatic and environmental suitability [3]; epidemiological and population-level determinants of dengue emergence; and [4] vector surveillance, control, and public health preparedness. Within each domain, findings were compared according to geographical setting, methodological approach, type of evidence, and relevance to Tehran. Particular emphasis was placed on whether a study provided evidence of an observed condition, inferred environmental suitability, a modeled future scenario, or an operational preparedness consideration. This distinction allowed the synthesis to maintain a clear separation between observed phenomena and projected or inferred risks [24], [25], [37], [38].

### 2.6 Qualitative synthesis and assessment of evidence relevance

Because of the substantial methodological, geographical, and contextual heterogeneity of the included evidence, synthesis was qualitative and integrative rather than quantitative. No meta-analysis, pooled effect estimate, formal statistical heterogeneity analysis, or inferential statistical testing was performed. Similarly, no original ecological niche model, climate–dengue transmission model, or predictive GIS analysis was developed within this review. Instead, evidence was compared narratively across the four predefined domains to identify areas of convergence, divergence, uncertainty, and evidence gaps. This approach was intended to characterize the pathway from environmental receptivity to potential vector establishment and, ultimately, possible dengue transmission without assuming that evidence supporting one stage automatically establishes the subsequent stage. A narrative assessment of confidence in the evidence was undertaken according to its geographical proximity to Tehran and methodological basis. Evidence derived directly from Tehran was considered more informative for local interpretation than evidence originating from other geographical settings. Evidence from Iran or geographically relevant neighboring settings was considered useful contextual evidence, while findings from comparable international environments were interpreted according to their ecological or climatic comparability. Validated regional or global modeling studies were interpreted as model-based evidence of potential climatic or spatial suitability and not as direct evidence of vector establishment or dengue transmission. Broad global projections were therefore interpreted more cautiously because of their greater geographical and ecological distance from Tehran. On this basis, the evidence concerning the absence of documented sustained autochthonous dengue transmission in Tehran was interpreted with greater confidence within the available evidence base than conclusions concerning future vector establishment or local transmission, for which direct longitudinal evidence remains limited [14], [24], [39].

### 2.7 Contextual environmental and meteorological assessment

Publicly available meteorological information from the Iran Meteorological Organization (IRIMO) was additionally reviewed descriptively to contextualize historical environmental conditions in Tehran, with particular attention to temperature, precipitation, and related climatic patterns relevant to *Aedes* ecology. These observations were used as contextual indicators of environmental suitability and were not treated as direct measurements of dengue transmission potential. No Tehran-specific dynamically downscaled climate–dengue transmission model was developed, and no causal attribution of future dengue incidence to climate change was attempted. Accordingly, climatic observations and published climate-based projections were interpreted as evidence concerning possible changes in environmental receptivity rather than as quantitative forecasts of future dengue transmission. Population-density information and urban land-use characteristics were likewise considered as contextual elements when interpreting potential breeding habitats, human–vector contact opportunities, and infrastructural or environmental vulnerability. These variables were not incorporated into a validated quantitative risk model, and no independent statistical estimates of their relative contributions to dengue emergence were generated. Urban environmental characteristics including housing conditions, water-management practices, potential artificial breeding habitats, waste accumulation, drainage conditions, and other relevant features were therefore considered complementary determinants of environmental receptivity rather than independently demonstrated predictors of *Aedes* establishment or dengue transmission [3], [13], [40], [41].

### 2.8 Conceptual and comparative visualization

Conceptual and descriptive figures were developed to integrate the principal evidence domains and illustrate relationships among climatic conditions, urban environments, *Aedes* ecology, virus introduction, surveillance, and public health preparedness. Microsoft Excel and QGIS were used for visualization and synthesis. These figures were designed to facilitate interpretation of heterogeneous evidence rather than to constitute original predictive models or validated quantitative risk-assessment instruments. Accordingly, any numerical scales presented in conceptual radar or comparative figures are illustrative and represent qualitative relative patterns identified in the reviewed literature rather than empirically calibrated measurements, validated risk scores, or estimates of absolute probability. The figures should therefore not be interpreted as demonstrating a quantitative level of dengue risk, vector establishment probability, or public health preparedness for individual Tehran districts. This clarification applies equally to comparative assessments derived from heterogeneous evidence sources and is intended to prevent visual representation from being mistaken for statistical estimation or formal validation [14], [24], [42].

### 2.9 Methodological boundaries

The methodological framework was deliberately designed to preserve the distinction between direct observations, contextual evidence, and model-based projections. The review did not assume that evidence of climatic suitability demonstrates *Aedes* establishment, that evidence of vector presence demonstrates persistence, or that imported dengue infection demonstrates autochthonous transmission. Similarly, recommendations concerning surveillance or preparedness were interpreted as evidence-informed considerations unless directly supported by published guidance or empirical evidence. The review itself did not generate or validate a specific trap density, surveillance frequency, district-level intervention threshold, or quantitative preparedness score for Tehran. The resulting methodology was therefore intended to provide a transparent and scientifically cautious synthesis of a heterogeneous evidence base rather than a quantitative prediction of dengue emergence. The principal analytical objective was to determine which conditions relevant to dengue emergence are supported by available evidence, how directly those conditions can be linked to Tehran, and where substantial uncertainty remains. This methodological approach provides the basis for interpreting the results while explicitly limiting extrapolation from regional or global evidence to the Tehran setting [18], [21].

## 3 Results

The evidence identified through the review converged across four interconnected domains relevant to dengue emergence risk in Tehran [1]: *Aedes* ecology and potential vector establishment [2]; climatic and environmental suitability [3]; epidemiological, population-level, urban, and infrastructural determinants; and [4] vector surveillance, control, and public health preparedness. The focused analytical synthesis comprised 12 core studies selected for their complementary contribution to these domains. The included evidence encompassed entomological and arbovirus-surveillance investigations from Iran, ecological studies conducted in temperate and urban settings, regional and global climate-risk and spatial modeling studies, and international reviews addressing dengue vector control and arboviral preparedness. The 12 studies differed substantially in geographical setting, study design, outcome measures, and evidentiary proximity to Tehran. Their principal characteristics, geographical contexts, study types, analytical focus, and specific contribution to the present synthesis are summarized in Table 1. Because of this heterogeneity, the findings were synthesized qualitatively and comparatively rather than quantitatively pooled. The reviewed evidence did not demonstrate sustained establishment of *Aedes aegypti* or *Aedes albopictus* populations in Tehran. Entomological investigations from Iran, including studies conducted outside the capital, documented mosquito fauna and ecological conditions relevant to *Aedes* biology, while arbovirus-surveillance studies emphasized the importance of systematic mosquito monitoring and laboratory-based surveillance. However, these findings did not provide direct evidence of persistent *Aedes* populations within Tehran. The Tehran-specific evidence available for the review lacked sufficiently standardized longitudinal sampling to establish temporal persistence, population continuity, or stable spatial distribution of either species. Differences among available studies in sampling strategies, surveillance intensity, geographical coverage, temporal repetition, and species-confirmation procedures further limited direct comparison across investigations. In particular, the available evidence did not provide a sufficiently consistent longitudinal framework incorporating standardized trap deployment, comparable sampling density, repeated larval surveillance, and species confirmation across time and locations in Tehran. Accordingly, the current evidence base supports an unconfirmed status of persistent *Aedes* establishment rather than evidence of established endemic vector populations [6], [33].

Across the evidence base, climatic variables emerged as important determinants of *Aedes* ecological suitability. International ecological and modeling studies identified temperature, precipitation, and related environmental variables as factors influencing mosquito geographic distribution, seasonal activity, and potential range expansion. Global climate-risk and spatial models included in the synthesis projected expansion of areas environmentally suitable for *Aedes* vectors or dengue-related transmission under changing climatic conditions. Ecological evidence from temperate urban environments also demonstrated that competent *Aedes* species can adapt to environmental conditions outside traditionally recognized tropical settings. These findings provided contextual evidence for assessing environmental receptivity in Tehran but did not constitute direct evidence of local vector establishment or dengue transmission. The Tehran-related climatic evidence indicated changes potentially relevant to mosquito ecology, particularly with respect to temperature and the duration of warm periods. The reviewed meteorological information and climate-related evidence suggested a warming tendency over the period considered, although the magnitude and characterization of climatic change varied according to the dataset, temporal interval, and analytical approach. Precipitation variability and seasonal climatic patterns were also considered potentially relevant environmental determinants. Urban environmental characteristics provided an additional layer of ecological heterogeneity, with temperature patterns, land use, housing conditions, water availability, artificial containers, water-storage practices, waste accumulation, and localized drainage conditions identified as factors that could modify the availability of mosquito breeding habitats. These findings collectively indicated that climatic and urban environmental conditions may contribute to environmental receptivity; however, no validated district-level estimate of *Aedes* establishment probability was available, and no Tehran-specific dynamically downscaled climate–dengue transmission model was developed in the present review. The epidemiological evidence reviewed did not demonstrate sustained autochthonous dengue transmission in Tehran. Evidence concerning dengue activity in Iran was more consistent with travel-associated or geographically introduced risk than with established endemic transmission in the capital. At the same time, population mobility and imported dengue infections represented plausible opportunities for virus introduction into an environment that could potentially become receptive to competent vectors. The reviewed evidence did not support interpreting imported dengue infection alone as evidence of subsequent local transmission. Population density, housing conditions, household and community water-storage practices, waste management, sanitation, and community or individual behaviors were identified as contextual factors that may influence mosquito breeding opportunities and human–vector contact. However, the available evidence did not provide sufficiently standardized Tehran-specific data to quantify the independent contribution of these factors to dengue emergence [13], [14].

The evidence concerning surveillance and preparedness indicated the importance of systematic entomological surveillance, epidemiological surveillance for imported dengue, appropriate laboratory capacity, vector identification, and coordinated response mechanisms. The available evidence also indicated that conventional mosquito-control activities may not necessarily provide sufficient sensitivity for early recognition of newly introduced or geographically restricted *Aedes* populations, particularly where surveillance has not been standardized longitudinally. The literature emphasized the value of integrating entomological surveillance with epidemiological and laboratory information so that changes in vector distribution, imported infections, and potential signals of local transmission can be recognized in a coordinated manner. Evidence from Iran and international arboviral preparedness literature therefore supported integrated surveillance as an important component of preparedness. The available evidence did not, however, provide sufficient operational data to quantify Tehran's overall preparedness, assign a validated preparedness score, or establish empirically derived city-wide thresholds for surveillance intensity, trap density, or intervention. Consequently, statements concerning surveillance fragmentation, operational limitations, or resource constraints were treated as evidence-informed areas requiring further assessment rather than as quantitatively demonstrated deficiencies across Tehran. The reviewed evidence instead identified several operational domains requiring integration, including longitudinal entomological monitoring, recognition of imported dengue infections, laboratory confirmation, environmental source reduction, vector-control capacity, and coordination among relevant public health and operational sectors. These findings were synthesized as components of preparedness rather than as evidence of a quantified preparedness deficit [18], [21].

When the evidence was considered across the four analytical domains, the strongest direct conclusion concerned the absence of documented sustained autochthonous dengue transmission and the lack of sufficient evidence to confirm persistent *Aedes aegypti* or *Aedes albopictus* establishment in Tehran. Evidence concerning future environmental receptivity was broader but generally less direct, comprising Tehran-related climatic observations, urban environmental characteristics, entomological evidence from other Iranian regions, ecological evidence from comparable urban settings, and regional or global climate and spatial modeling. These evidence streams converged on conditions that may influence *Aedes* suitability and the potential for dengue emergence but differed substantially in geographical proximity and methodological basis. The evidence structure therefore did not support a quantitative estimate of future dengue transmission risk in Tehran. Instead, it demonstrated a graded evidence pattern in which direct evidence regarding current local transmission and persistent vector establishment remained limited, while indirect evidence concerning environmental and climatic receptivity was comparatively broader. Overall, the findings support characterization of Tehran as a setting in which conditions relevant to dengue emergence may warrant continued assessment, rather than as a city with demonstrated established dengue transmission. The evidence did not demonstrate that environmental suitability, climatic change, population mobility, or isolated ecological observations had progressed to sustained vector establishment or autochthonous dengue transmission. Similarly, the available evidence did not permit determination of whether a transition from environmental receptivity to persistent vector establishment is occurring. The principal empirical pattern emerging from the synthesis was therefore the coexistence of limited direct evidence for current local transmission with a broader body of contextual evidence indicating potentially relevant ecological, climatic, epidemiological, and preparedness conditions (Table 2) [4], [14].

**Table 2:** Evidence domains, evidence directness, and key findings relevant to dengue emergence risk in Tehran.

Evidence domain	Evidence directness to Tehran	Key evidence and findings	Interpretation for dengue emergence risk in Tehran
Aedes ecology and potential establishment	Direct Tehran evidence: limited; supported by Iranian and regional entomological evidence	Available evidence does not provide sufficient longitudinal, standardized, and species-confirmed data to demonstrate sustained establishment of *Aedes aegypti* or *Aedes albopictus* in Tehran. Entomological evidence from other parts of Iran documents mosquito fauna and environmental conditions relevant to *Aedes* ecology, but Tehran-specific evidence remains limited in temporal coverage, sampling standardization, geographic coverage, and species confirmation.	Persistent establishment of *Aedes aegypti* or *Aedes albopictus* in Tehran remains unconfirmed. Available evidence supports continued standardized and longitudinal entomological surveillance rather than assuming established vector populations.
Climatic suitability and climate variability	Indirect to moderate; based mainly on regional/global studies and descriptive Tehran climate evidence	Warming, changes in the duration of warm periods, precipitation variability, and urban climatic conditions may modify environmental suitability for *Aedes* ecology. Global and regional climate-risk and spatial models indicate that climate change may expand areas environmentally suitable for *Aedes* vectors and dengue-related transmission.	Climate change may contribute to increased environmental receptivity, but climatic suitability alone cannot demonstrate local vector establishment or dengue transmission in Tehran. Model-based projections should therefore be interpreted as potential changes in suitability rather than predictions of local transmission.
Urban environmental determinants	Tehran-specific contextual evidence: moderate; quantitative evidence limited	Urban density, housing characteristics, household and community water storage, waste accumulation, drainage conditions, land-use patterns, and other environmental characteristics may create localized conditions favorable to mosquito breeding and persistence. The urban heat-island effect may further modify local temperature conditions.	Urban environmental characteristics may modify localized environmental receptivity, but their relative contribution has not been quantified reliably and no validated district-level estimate of *Aedes* establishment probability is currently available for Tehran.
Population mobility and virus introduction	Iran/Tehran relevance: moderate; direct transmission evidence limited	International and domestic population mobility may introduce dengue virus into areas where competent vectors are present or subsequently become established. Imported dengue infections represent an opportunity for viral introduction but do not, by themselves, demonstrate local transmission.	Population mobility represents a potential pathway for virus introduction and may become increasingly relevant if competent vectors establish persistent populations. Imported infection should not be interpreted as evidence of autochthonous transmission.
Dengue epidemiology and autochthonous transmission	Direct Tehran evidence: limited but directionally consistent	The reviewed evidence does not demonstrate sustained autochthonous dengue transmission in Tehran. Available dengue activity in Iran is more consistent with travel-associated or geographically imported risk than with established endemic transmission in the capital.	Tehran should currently be regarded as a setting of potential dengue emergence rather than established dengue transmission. The absence of documented sustained transmission does not exclude future risk.
Social and infrastructural determinants	Contextual/indirect; Tehran-relevant but not independently quantified	Water-management practices, sanitation, housing conditions, environmental maintenance, waste management, public awareness, community practices, and individual behavior may influence the availability of breeding habitats and opportunities for human–vector contact. Their effects may interact with climatic and urban conditions.	These factors may modify local receptivity and exposure, but their individual and relative contributions to dengue emergence risk in Tehran remain uncertain and insufficiently quantified.
Vector surveillance capacity	Tehran-specific evidence: limited	Available evidence indicates a need for more systematic and longitudinal surveillance capable of detecting *Aedes* presence, persistence, seasonal dynamics, population density, and geographic expansion. Existing evidence is insufficient to determine long-term population persistence across Tehran.	Strengthening standardized entomological surveillance is important for distinguishing sporadic or transient detection from sustained vector establishment and for reducing uncertainty regarding the current vector status of Tehran.
Dengue and arbovirus epidemiological surveillance	Iran/Tehran relevance: moderate	Integration of epidemiological, entomological, environmental, and laboratory surveillance is important for early detection of imported dengue infections and possible local transmission. Evidence from Iran and international preparedness literature emphasizes systematic surveillance, laboratory capacity, early recognition, and coordinated response to emerging arboviral threats.	Integrated surveillance can reduce uncertainty and enable earlier detection of a transition from virus introduction to possible local transmission, particularly if competent vectors become established.
Vector-control preparedness	Contextual evidence: moderate; operational effectiveness in Tehran not quantitatively evaluated	Conventional mosquito-control approaches may require adaptation to the ecology of *Aedes* mosquitoes, particularly in urban environments where breeding habitats can occur around households and human activity. The reviewed evidence supports integrated and preparedness-oriented approaches involving early detection, targeted investigation, environmental source reduction, and coordinated response.	Preparedness should emphasize early detection and evidence-based response rather than assume an established outbreak. No validated Tehran-specific operational threshold, trap-density target, or preparedness score was established by this review.
Global and regional dengue-risk models	Indirect; model-based evidence	Climate-driven and spatial models indicate potential expansion of *Aedes* climatic suitability and populations living in areas potentially suitable for dengue transmission under changing environmental conditions. These models generally operate at broader geographic and ecological scales than Tehran.	Model-based evidence supports future risk assessment and preparedness, but cannot be interpreted as evidence of current *Aedes* establishment or future dengue transmission in Tehran. The uncertainty associated with transferring broad-scale model projections to Tehran must be retained.
Overall evidence synthesis	Mixed: direct Tehran evidence limited; Iranian, regional, and global evidence complementary	The available evidence converges on a combination of climatic and urban environmental factors that may increase environmental receptivity, together with population mobility that may provide opportunities for virus introduction. However, evidence for sustained *Aedes* establishment and autochthonous dengue transmission in Tehran remains insufficient.	Overall, Tehran is best characterized as a setting of potential and evolving dengue receptivity, rather than an established dengue-endemic setting. The principal public health implication is strengthened surveillance and preparedness, not an assumption of imminent endemic transmission.

**Table note:** Evidence directness refers to the geographical and inferential proximity of each evidence domain to Tehran. Direct Tehran evidence was distinguished from evidence obtained from other Iranian regions, comparable international settings, and regional or global modeling studies. Model-based evidence was interpreted as evidence of potential climatic or spatial suitability and **not** as evidence of vector establishment or dengue transmission. Environmental receptivity, vector establishment, and sustained autochthonous transmission represent distinct stages of the emergence pathway and should not be treated as interchangeable outcomes. The review did not identify sufficient evidence to calculate a validated quantitative dengue-risk or public health preparedness score for Tehran; therefore, surveillance and preparedness implications are presented as evidence-informed considerations rather than validated operational thresholds.

## 4 Discussion

The present integrative review indicates that dengue emergence risk in Tehran should be interpreted as a multidimensional and evolving process rather than as a consequence of any single climatic, entomological, or epidemiological determinant. The principal finding is that the available evidence does not demonstrate sustained autochthonous dengue transmission or provide sufficient evidence to confirm persistent establishment of *Aedes aegypti* or *Aedes albopictus* in Tehran. At the same time, the evidence identifies several conditions that may contribute to environmental receptivity, including climatic variability and warming, urban environmental characteristics, potential breeding-site availability, population mobility, regional entomological observations, and the capacity of surveillance and response systems to detect emerging arboviral threats. Importantly, these components represent different levels of inference and should not be treated as interchangeable. The evidence supporting the current absence of documented sustained local transmission is stronger and more direct than the evidence available for predicting future vector establishment or dengue transmission. The appropriate interpretation is therefore one of potential and evolving receptivity, accompanied by substantial uncertainty regarding whether the ecological pathway from environmental suitability to persistent vector establishment and subsequently to local transmission is occurring in Tehran [14], [18].

A central implication of the findings is the need to distinguish clearly between environmental receptivity, vector establishment, and dengue transmission. Environmental receptivity refers to the presence of climatic, ecological, urban, and social conditions that could permit a competent vector to survive and reproduce if introduced. Establishment represents a substantially stronger biological condition, requiring evidence that a vector population can persist and reproduce over time within an appropriate spatial context. Sustained dengue transmission requires an additional sequence of events, including introduction of dengue virus, adequate vector abundance and competence, sufficient human–vector contact, and temporal and spatial overlap among the virus, competent vectors, and susceptible human populations. The evidence synthesized here does not demonstrate that these conditions currently coexist in Tehran ((Fig. 2). Consequently, evidence of climatic suitability, favorable urban microhabitats, or potential breeding environments should not be interpreted as evidence of established *Aedes* populations, and evidence of imported dengue infection should not be interpreted as evidence of autochthonous transmission. Maintaining this distinction is essential for avoiding overestimation of current dengue risk while still recognizing that environmental conditions may change the future receptivity of the city. The uncertainty surrounding *Aedes* establishment in Tehran is particularly important. Entomological evidence from Iran and other settings demonstrates that mosquito ecology can vary substantially according to local environmental conditions, while studies from temperate urban environments show that *Aedes* species can persist outside traditionally recognized tropical settings when ecological conditions are suitable. Nevertheless, evidence generated in southern Iran, other Iranian regions, or international urban environments cannot substitute for standardized longitudinal surveillance in Tehran. The available Tehran-specific evidence does not provide sufficiently consistent information on temporal persistence, spatial continuity, population density, repeated larval occurrence, or molecularly confirmed species identification to establish that *Aedes aegypti* or *Aedes albopictus* populations are persistently established in the city. Differences in trapping approaches, sampling intensity, geographical coverage, duration of surveillance, and species-confirmation procedures further constrain comparison among studies. Thus, the current evidence should not be interpreted either as confirmation of vector establishment or as definitive proof that transient introductions or localized populations cannot occur. Rather, the principal conclusion is that the establishment status remains insufficiently characterized [3], [35].

**Fig. 2:**
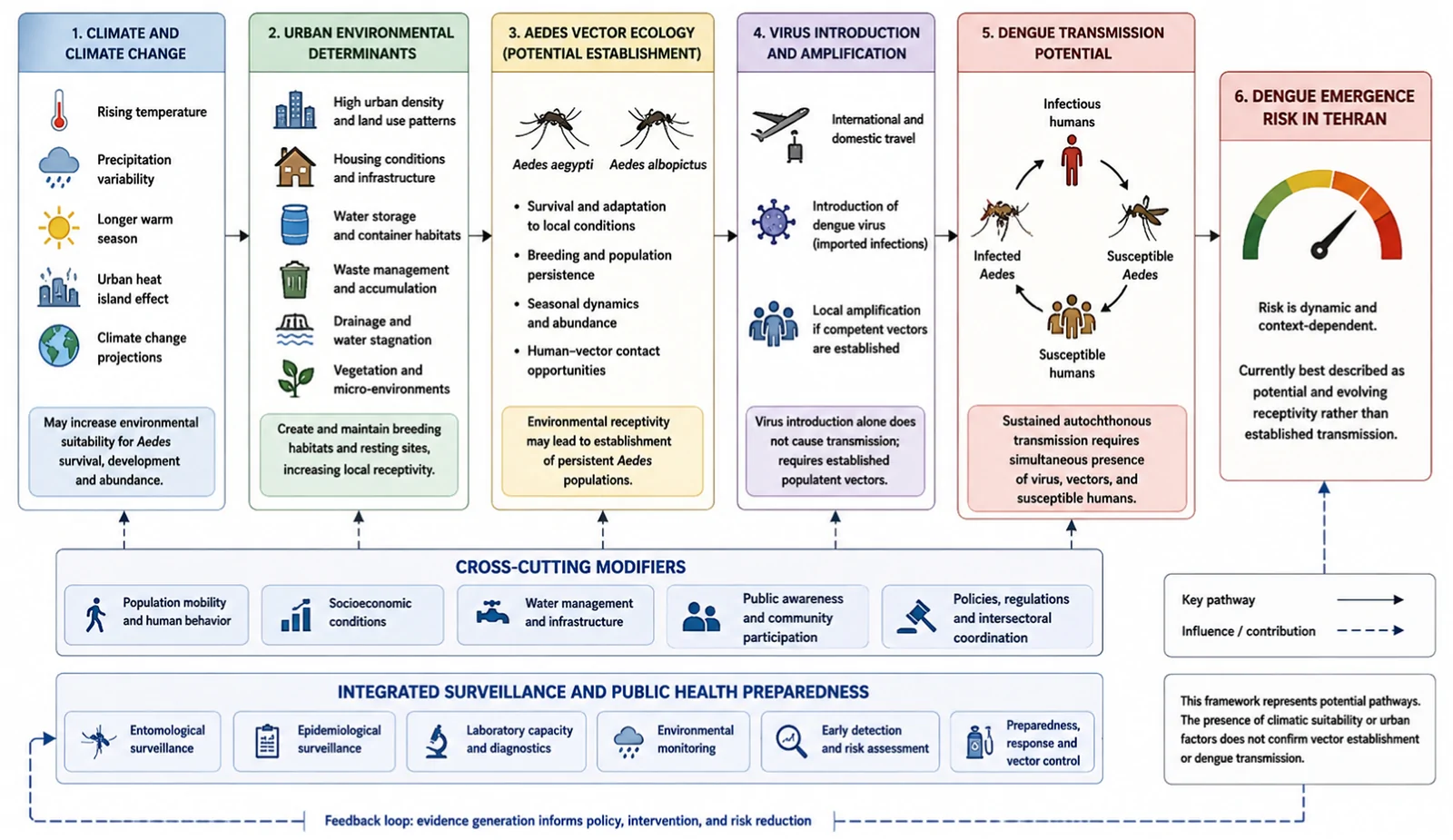
Conceptual framework linking climate, urban environment, *Aedes* vector ecology, virus introduction, and dengue emergence risk in Tehran. This conceptual framework illustrates the potential pathways through which climatic conditions and climate variability, urban environmental determinants, *Aedes* vector ecology, and population mobility may interact to influence environmental receptivity and the potential for dengue emergence in Tehran. Climatic and urban conditions may affect the suitability of habitats for *Aedes aegypti* and *Aedes albopictus*, including vector survival, breeding-site availability, seasonal persistence, and human–vector contact; however, environmental suitability does not demonstrate persistent vector establishment. Virus introduction through imported infections may create opportunities for local amplification only if competent and sufficiently abundant vectors and susceptible human populations are simultaneously present. Sustained autochthonous transmission therefore requires the convergence of virus introduction, established vector populations, and adequate human–vector contact. Cross-cutting social, infrastructural, and behavioural determinants, together with integrated entomological, epidemiological, laboratory, and environmental surveillance, influence both emergence potential and the capacity for early detection and response. The framework is conceptual and does not represent a validated quantitative risk model; the presence of climatic or urban suitability should not be interpreted as evidence of established *Aedes* populations or current autochthonous dengue transmission in Tehran.

The climatic evidence provides a biologically plausible basis for considering changing environmental receptivity, but its interpretation requires particular caution. The reviewed international and regional studies consistently support the role of temperature, precipitation, seasonal conditions, and other environmental variables in determining *Aedes* distribution and dengue-related ecological processes. Climate-based and spatial models also indicate that warming may alter the geographical extent of environments suitable for *Aedes* vectors or dengue transmission. However, these studies operate at broader geographical and methodological scales than the specific question of whether dengue transmission will occur in Tehran. A modeled increase in climatic suitability represents a change in environmental conditions that may facilitate vector survival or transmission under appropriate biological circumstances; it does not establish that a competent vector has become established locally, nor does it demonstrate that dengue virus will subsequently circulate among humans. Accordingly, the model-based literature is best interpreted as contextual evidence for potential changes in environmental receptivity rather than as a Tehran-specific prediction of future dengue incidence. The absence of a Tehran-specific dynamically downscaled transmission model and validated district-level establishment estimates further limits the precision with which future risk can currently be characterized. Climate, moreover, should not be considered independently of the urban environment. The evidence reviewed suggests that housing characteristics, water-storage practices, drainage conditions, waste accumulation, land use, and other features of the built environment may influence the availability and persistence of mosquito breeding habitats. Urban temperature patterns, including localized heat effects, may further modify the ecological conditions experienced by mosquitoes. These determinants are spatially heterogeneous and may interact with climatic conditions, meaning that broad city-level climatic trends cannot necessarily be translated into uniform vector suitability across Tehran. Nevertheless, the present evidence does not support a validated district-level model capable of identifying specific neighborhoods as having a defined probability of *Aedes* establishment. The appropriate interpretation is therefore that urban environmental characteristics constitute plausible modifiers of local receptivity whose magnitude and spatial distribution remain insufficiently quantified. Future assessments should consequently move beyond city-wide climatic averages and integrate entomological observations with appropriately resolved information on land use, water management, housing, environmental maintenance, and other relevant urban characteristics [13], [14].

Population mobility represents another component of the emergence pathway, but it should likewise be interpreted as a mechanism for potential virus introduction rather than evidence of transmission. Imported dengue infections can create opportunities for virus introduction into a population, particularly if competent vectors are present or subsequently establish. However, introduction alone is insufficient for sustained local transmission because it must coincide with competent and sufficiently abundant vectors, appropriate environmental conditions, and adequate human–vector contact. The evidence available for Tehran therefore supports consideration of mobility as a potential epidemiological bridge between external dengue activity and local emergence, but does not demonstrate that this bridge currently results in sustained transmission. This distinction is particularly important when interpreting dengue activity reported elsewhere in Iran or among travelers, because geographical or travel-associated infection may increase opportunities for introduction without demonstrating establishment of an endemic transmission cycle in Tehran. The findings also have implications for how surveillance and preparedness should be conceptualized. The evidence supports integration of longitudinal entomological surveillance with epidemiological surveillance for imported dengue, laboratory capacity for reliable pathogen and vector identification, and mechanisms for coordinated public health response. Such integration is particularly relevant where the principal uncertainty concerns whether occasional vector detection or virus introduction represents an isolated event or the early stage of a sustained ecological process. Early recognition of changes in vector distribution, seasonal persistence, or imported infection patterns may reduce the time between detection and appropriate response. However, the present review does not provide sufficient operational evidence to conclude that Tehran has a specific quantified level of preparedness, nor does it support assigning a validated preparedness score to the city. Similarly, the review does not establish an empirically validated trap density, surveillance frequency, district-level intervention threshold, or another numerical operational target. Accordingly, any proposed surveillance intensity or operational threshold should be identified explicitly as an evidence-informed operational consideration unless it is supported by a published guideline or locally validated empirical study (Fig. 3) [18], [21].

**Fig. 3:**
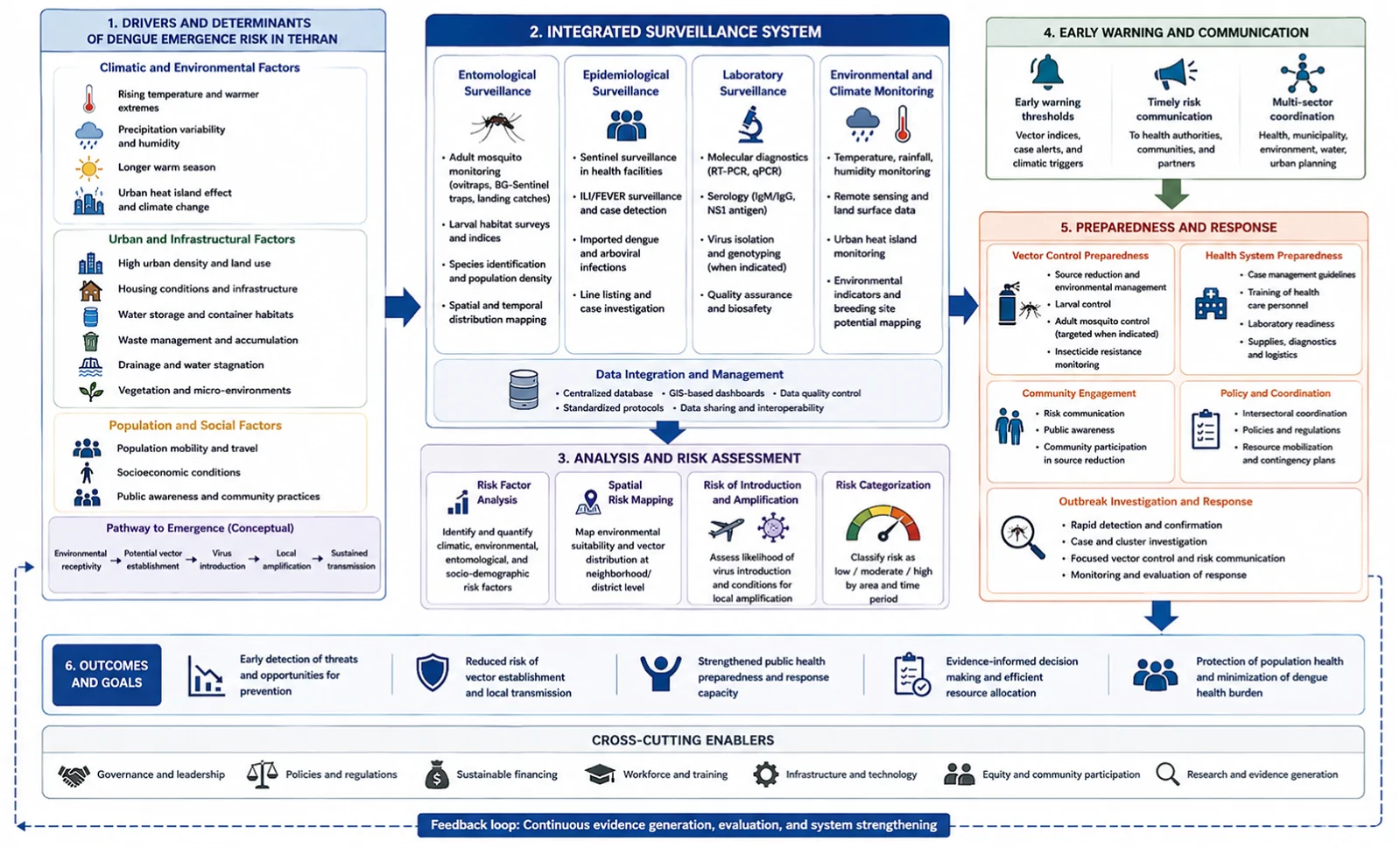
Integrated framework of vector surveillance and public health preparedness for potential dengue emergence in Tehran. This framework integrates the major environmental, urban, infrastructural, population, and social determinants that may influence dengue emergence risk in Tehran with an interconnected surveillance and preparedness system. The proposed framework links entomological surveillance of adult and immature *Aedes* populations, epidemiological surveillance of suspected and imported dengue and other arboviral infections, laboratory confirmation and molecular characterization, and environmental and climate monitoring through coordinated data integration and analysis. These components support evidence-informed assessment of spatial and temporal patterns, environmental and vector-related risk factors, potential virus introduction, and conditions for local amplification, followed by early warning, risk communication, vector-control preparedness, outbreak investigation, health-system readiness, community engagement, and intersectoral coordination. The framework emphasizes continuous feedback between surveillance, risk assessment, preparedness, response, and evidence generation to strengthen public health capacity. It is a conceptual and operational framework rather than a validated quantitative risk or preparedness index; therefore, the proposed surveillance components, thresholds, and response actions should be interpreted as evidence-informed considerations rather than empirically validated Tehran-specific thresholds.

The role of the 12 core studies should also be interpreted in light of the heterogeneous evidence base. These studies were not selected because they constituted a statistically homogeneous sample from which a pooled estimate of dengue emergence risk could be calculated. Rather, they were selected because, collectively, they provided complementary information across the principal domains of the review: Iranian entomological and arbovirus-surveillance evidence contributed geographically relevant information; ecological studies from comparable urban and temperate environments informed interpretation of vector adaptation and establishment; climate and spatial modeling studies contributed evidence concerning potential changes in environmental suitability; and international public health literature informed interpretation of surveillance, vector control, and preparedness. This approach allowed the review to connect evidence generated at different geographical scales while explicitly preserving the distinction between direct and indirect evidence. The resulting synthesis is therefore informative at the level of mechanism, context, and preparedness, but it should not be interpreted as a quantitative estimate of the probability of dengue emergence in Tehran. The heterogeneity of the 12 studies is a limitation for quantitative inference but also reflects the multidisciplinary nature of the research question addressed by this review [24], [43].

### 4.1 Strengths and limitations

This review has several strengths. First, it addresses dengue emergence as a coupled ecological, climatic, epidemiological, urban, and public health problem rather than reducing risk assessment to climate suitability alone. Second, it explicitly separates evidence according to its geographical and inferential proximity to Tehran, distinguishing Tehran-specific observations from evidence derived from other Iranian regions, comparable international settings, and regional or global models. Third, the focused synthesis of 12 core studies provides a transparent structure for integrating heterogeneous evidence while avoiding inappropriate quantitative pooling. Fourth, the interpretation does not treat the absence of documented vector establishment as definitive evidence that vectors cannot be introduced or temporarily detected; instead, it recognizes the limitations created by insufficient standardized longitudinal surveillance. Fifth, the synthesis links environmental receptivity with the conditions required for vector establishment, virus introduction, and sustained transmission, thereby providing a framework that is more consistent with the multistage nature of dengue emergence. This structured approach is particularly important for a narrative/integrative review because transparent linkage between the underlying evidence, synthesis, and conclusions is essential when statistical pooling is not appropriate. Several limitations should nevertheless be considered when interpreting the findings. The most important is the limited availability and heterogeneity of Tehran-specific longitudinal entomological evidence. Existing studies vary in surveillance methodology, geographical coverage, temporal duration, sampling intensity, and species-confirmation procedures, restricting the ability to distinguish transient introductions, localized occurrence, and sustained population establishment. A second limitation is the use of evidence from other Iranian regions, comparable international urban settings, and regional or global modeling studies to contextualize Tehran. Although these sources provide biologically and epidemiologically relevant information, they cannot replace locally generated longitudinal evidence. A third limitation is the qualitative nature of the synthesis: no meta-analysis, pooled effect estimate, formal statistical heterogeneity analysis, or inferential statistical modeling was undertaken. A fourth limitation is that the review did not develop an original ecological niche model, predictive GIS model, or Tehran-specific climate–transmission model; consequently, it cannot provide a validated numerical estimate of future vector establishment or dengue transmission risk. A fifth limitation concerns the conceptual figures and comparative assessments used to organize the evidence. These were developed as descriptive or conceptual synthesis tools and were not validated risk, receptivity, or preparedness indices; any numerical scales used in such visualizations should therefore be understood as illustrative rather than empirically calibrated measurements. Finally, because the review was designed as a structured narrative/integrative synthesis rather than a conventional systematic review and was not prospectively registered, its methodological framework does not provide the same formal risk-of-bias and quantitative certainty assessments available in conventional systematic evidence syntheses. These limitations reinforce the need to interpret the findings as an evidence-informed assessment of potential emergence pathways rather than as a predictive model of future dengue transmission [24], [43], [44].

### 4.2 Conclusions

The findings of this review support a cautious, preparedness-oriented interpretation of dengue emergence risk in Tehran. Current evidence does not demonstrate sustained autochthonous dengue transmission or confirmed persistent establishment of *Aedes aegypti* or *Aedes albopictus* in the city. At the same time, climatic and urban environmental conditions, population mobility, regional entomological evidence, and findings from international ecological and climate-based studies identify a set of circumstances that may influence future environmental receptivity. These findings should not be interpreted as evidence that dengue transmission is imminent or inevitable. Rather, they indicate that the transition from environmental receptivity to vector establishment and subsequently to local transmission remains an unresolved empirical question. The most appropriate public health response is therefore not to assume endemic transmission, but to reduce uncertainty through standardized longitudinal entomological surveillance, integration of vector and epidemiological monitoring, improved characterization of relevant urban environmental determinants, and appropriate laboratory capacity for vector and pathogen identification. Such locally generated evidence would provide a stronger basis for determining whether changes in environmental receptivity are translating into persistent vector populations or local transmission and would reduce reliance on extrapolation from geographically distant ecological observations and broad-scale climate models [18], [21].

## Ethics statement

Not applicable. This study was conducted as a structured narrative/integrative review based exclusively on previously published literature and publicly available information from bibliographic databases and institutional sources. It did not involve the recruitment or participation of human subjects, the use of human biological materials, animal experimentation, clinical interventions, or the collection of identifiable personal or confidential participant data. No new primary data were collected from human or animal participants. Accordingly, formal ethical approval from an institutional research ethics committee and informed consent to participate were not required for this study.

## Data availability statement

The datasets generated and/or analyzed during the current study are available within the article and its supplementary materials. No additional datasets were generated.

## Consent for publication

Not applicable.

## Funding

This research received no specific grant from any funding agency in the public, commercial, or not-for-profit sectors.

## Declaration of competing interest

The authors declare no competing interests.
